# Fluctuating Neurological Deficits in a Patient With Sporadic Hemiplegic Migraine

**DOI:** 10.7759/cureus.78262

**Published:** 2025-01-30

**Authors:** Tavon Naddaf, Gurinder Ghotra

**Affiliations:** 1 Internal Medicine, Touro University California, Vallejo, USA; 2 Internal Medicine, San Joaquin General Hospital, French Camp, USA

**Keywords:** fluctuating symptoms, fluctuation, hemiplegic migraine, migraine with aura, postictal paralysis, sporadic hemiplegic migraine, todd's paralysis

## Abstract

This article aims to present and discuss a case of a patient presented with stroke-like symptoms that fluctuated with rapid initial improvement followed by recurrence and slow resolution over a period of six days. A 57-year-old male with a past medical history of chronic myeloid leukemia (CML), unspecified seizure disorder, hyperlipidemia (HLD), hypertension (HTN), peptic ulcer disease (PUD), and type II diabetes mellitus (T2DM) presented with right-sided focal neurological deficits (FNDs). The patient initially called the ambulance for intractable abdominal pain with a five-day history of melena and one episode of hematemesis followed by a ground-level fall, during which he was witnessed by family shaking and unresponsive. He was alert and oriented when he developed FNDs en route to the hospital. The initial differential included stroke, transient ischemic attack (TIA), seizure followed by Todd's paralysis (TP), and hemiplegic migraine (HM). The clinical picture was complex; the patient's history of T2DM, HLD, and HTN all placed him at high risk of stroke and the witnessed seizure suggested TP. Computed tomography (CT) and magnetic resonance imaging (MRI) were crucial in eliminating stroke as a possible etiology, and TIA was ruled out as symptoms persisted. Imaging, clinical findings, investigation of the patient's past medical history, and critical reasoning helped rule out TP. This was finally diagnosed and treated as a case of sporadic HM with unexpected fluctuations in aura. The fluctuation of symptom intensity raises questions about the pathophysiology of migrainous aura, how it produces FNDs, and how it can explain the presentation of this patient.

## Introduction

Focal neurological deficits (FNDs) are usually a sign of central nervous system (CNS) pathology, which can be roughly localized by physical examination and more definitively located by advanced imaging techniques, such as magnetic resonance imaging (MRI), MRI with fluid-attenuated inversion recovery (FLAIR), and computed tomography (CT) [1,2]. Because acute FNDs are the cardinal sign of cerebrovascular accident (CVA), even the earliest signs of facial droop, extremity weakness, numbness, and slurring of speech are reasons for emergent evaluation and imaging [1,2]. Some other deficits include altered consciousness, confusion, hemianopia, gaze palsy, dizziness, vertigo, aphasia, dysarthria, and dysphagia [3]. The American College of Radiology recommends a CT head without intravenous (IV) contrast, a CT angiography (CTA) head with IV contrast, a CTA neck with IV contrast, and an MRI head without IV contrast as the usual appropriate initial imaging in a patient presenting with FNDs clinically suspected of acute ischemic stroke [2].

However, FNDs are not unique to CVA, and many different pathologies can present with similar symptoms but varying imaging and clinical findings [1]. Chief among these is the closely related transient ischemic attack (TIA), a condition in which ordering imaging is appropriate but will show no acute findings [2]. TIA should resemble a known stroke syndrome due to its common nature, but there is no evidence of tissue damage on imaging [3,4]. There is no time constraint for TIA, although traditionally the limit was 24 hours and most cases are resolved in under one hour [2].

Post-ictal deficits following a seizure can resemble a stroke, especially Todd's paralysis (TP) [4]. TP is focal motor weakness that follows a seizure and was originally described as lasting up to 48 hours [5] although longer cases have been reported [4]. Initial imaging with diffusion-weighted MRI (DWI) can show increased cortical signal in the epileptogenic region [1], as well as increases in signal on T2 and FLAIR sequences representing cortical swelling [5].

Hemiplegic migraine (HM) is a subset of migraine with aura (MA), which can appear in familial or sporadic forms [6]. The symptoms are similar to MA except that there must be fully reversible motor weakness and sensory or speech symptoms [6]. Aura usually lasts an hour in MA but may persist much longer in HM [4,6]. Imaging is essential to separate this condition from CVA; there are no acute findings on T2, FLAIR, or DWI [1]. Migraine patients, however, do have an increased prevalence of white matter hyperintensities in the deep cortical areas independent of cardiovascular risk factors [7]. This is the condition the patient was diagnosed with and treated for.

## Case presentation

**Figure 1:**
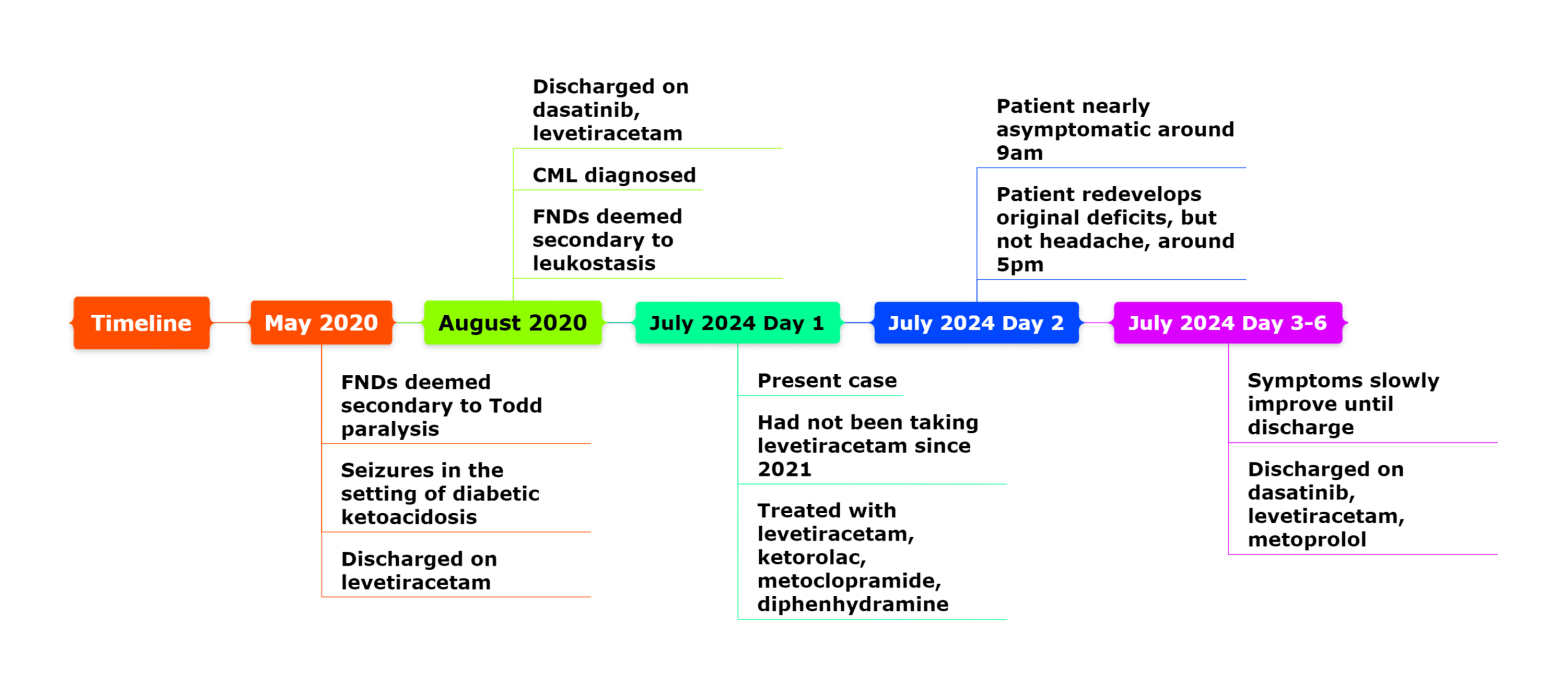
Timeline of the patient Created with Mindomo software (Expert Software Applications, Romania)

This is a 57-year-old male with a past medical history of chronic myeloid leukemia (CML), seizure disorder, peptic ulcer disease (PUD), hyperlipidemia (HLD), hypertension (HTN), and type II diabetes mellitus (T2DM). He presented to the emergency department (ED) as a patient with stroke-like symptoms. This patient has had similar presentations at least three times, first in May 2020, then in August 2020, and then in July 2024 (see Figure 1). This history will be discussed now, in brief, to give context to the case.

In May 2020, he presented to the ED with abdominal pain, hematemesis, and melena. He was found to be in diabetic ketoacidosis (DKA) with positive ketones and an elevated anion gap, which was treated accordingly. The hematemesis and melena were thought related to his history of PUD and ceased with proton pump inhibitor (PPI) therapy. He was rushed to the intensive care unit after suddenly developing agonal breathing, hypoxia on 10 L oxygen, and sustaining repeated seizures. He was intubated and treated with multiple doses of lorazepam. He was extubated within 24 hours but had focal weakness and visual loss after the seizure that lasted about two days. MRI was unremarkable except for deep cortical white matter hyperintensities that appeared benign. This was treated as TP and he was discharged on levetiracetam due to a newly diagnosed seizure disorder. This was also the episode CML was first suspected due to the incidental finding of leukocytosis with a white blood cell (WBC) count of 196k and a bone marrow biopsy suspicious for a myeloproliferative disorder. It is unknown why this was not addressed before August 2020.

The second presentation in August 2020 was for asymptomatic leukocytosis with a WBC count of 249k. Genetic testing for CML was positive. He was admitted to the hospital for leukapheresis and therapy with dasatinib, hydroxyurea, allopurinol, and rasburicase. On the third day of treatment, he had an episode of unresponsiveness for which their differential included acute cerebrovascular accident (CVA), seizure, and syncope (no further clarification exists). He was intubated for <24 hours, but after extubation, he had an episode of significant right-sided weakness and facial droop. Leukapheresis was repeated and the patient had significant improvement in neurologic symptoms shortly after treatment. Given significant clinical improvement after leukapheresis, it was judged his acute decompensation and transient right-sided weakness were related to a left middle cerebral artery TIA syndrome from leukostasis. This was judged atypical given he had CML and WBC in the 200k range without excess blasts. All imaging and an electroencephalogram (EEG) were unremarkable, except for a diffuse abnormal signal of the calvarium on MRI deemed secondary to leukemic involvement. Because of the possibility of seizure disorder (despite negative EEG), he was discharged on levetiracetam.

The patient was kept on dasatinib and his CML was effectively kept in remission since late 2020. His primary care physician discontinued the levetiracetam sometime in 2021-2022 because the diagnosis of epilepsy was doubtful; both seizures were provoked. He had several more visits to the ED, often for melena and hematemesis. The GI bleeding was treated as PUD and resolved with PPIs each time, but it is unclear whether testing and eradication of *Helicobacter pylori *was ever done or if the patient completed a full course of PPIs between episodes. All information for these episodes comes from hospital records. The patient recalled right-sided weakness, facial pain, and visual loss with blue spots similar to this episode, but this may be subject to recall bias. There are no further records of seizures or stroke symptoms until 2024.

In this case, the patient called the ambulance for intractable abdominal pain. The patient noted pain of intensity 8/10 associated with melenic diarrhea for the past five days and hematemesis that morning. He also reported dizziness followed by a ground-level fall. At that time he felt he was conscious, however, he was witnessed by his family unresponsive and shaking for almost one minute. He then called the ambulance. He denied confusion or drowsiness after the fall, odd for a suspected seizure. In the ambulance, he suddenly developed right-sided weakness, retro-orbital facial pain, and dancing blue spots in the visual field. He also reported photophobia and blurred vision in the right eye. The patient stated he was taking dasatinib for CML and insulin for diabetes. He denied any history of seizures, migraine, stroke, or a family history of medical illness. He was admitted to the hospital for further workup, treatment, and monitoring.

The patient was found to be awake, alert, in acute distress, and following commands. Initial National Institutes of Health Stroke Scale was 13. Vital signs are given in Table 1.

**Table 1:** Initial vital signs °C = degrees Celsius, bpm = heart beats per minute, mmHg = millimeters mercury, rpm = respirations per minute

Measurement	Patient's value	Reference range
Temperature	36.6 °C	36.5-37.5 °C
Heart rate	77 bpm	60-100 bpm
Systolic blood pressure	177 mmHg	<130 mmHg
Diastolic blood pressure	93 mmHg	<80 mmHg
Respiratory rate	20 rpm	12-20 rpm
Oxygen saturation	96%	95-100%

He was dysarthric but with no visible deviation of tongue or uvula. Left-sided monocular vision, strength, sensation, reflexes, and tone were completely normal. The left face was likewise normal in strength, tone, and sensation.

The following findings are entirely right-sided. He had monocular vision loss, notable facial asymmetry with absent forehead wrinkles, and nasolabial fold along with complete paralysis. The eye, however, was shut closed and could not be opened voluntarily. Curiously, the patient was also unable to force this eye closed when resisted. Reports of photophobia and vision loss were obtained with the eye held open. Extremity strength was 0/5 with no effort against gravity and no sign of muscle contraction. The muscles had a flaccid tone. Patellar, Achilles, and bicipital reflexes were absent and Babinski's sign was negative. There was a loss of sensation to pinprick and light touch on the face, body, and extremities. The physical examination shed doubt on an ischemic etiology, where one might expect an eye trapped open, spasticity, hyperreflexia, and a positive Babinski sign. This distribution of FNDs was also not consistent with a known stroke syndrome.

The abdomen was non-distended with no masses, but there was tenderness to palpation with guarding in the left lower quadrant. This was not where one would expect to find tenderness in PUD. The patient vomited nonbloody mucus during physical examination. Blood pressure and respiratory rate normalized once the pain was controlled.

By day two, his face was symmetrical and he was able to raise his right arm without impediment and hold his right leg against gravity for almost five seconds. Patellar and bicipital reflexes were normal on the left and slightly increased on the right. Babinski's sign was negative bilaterally. The blurry vision and dancing blue spots were unchanged but he denied headache. The patient also denied abdominal pain, hematemesis, or melena as no stools had passed. On afternoon rounds, it was observed that the face was drooping again with the right eye closed and the right arm and leg unable to challenge gravity. The headache, however, did not recur. He was alert and oriented, although distressed that his symptoms had worsened after an initial promising improvement. He did not have any seizure-like episodes before this recurrence, which weakened the argument for TP causing his initial presentation.

After the sudden recurrence on the afternoon of day two, symptoms continued to improve. Weakness and loss of sensation improved gradually. Reflexes were normal on day three. Photosensitivity, blurred vision, and blue scotoma remained until day five. He was able to walk with assistance by day four, and on his own by day six. Stools were brown and of normal consistency throughout hospitalization. Physical therapy recommended short-term rehabilitation because he walked with a minor limp, but the patient declined. He stated he would rather continue recovery at home than stay in a clinical setting and accepted that this might prolong his recovery. He was discharged on day six.

Diagnostic assessment

Table 2 shows significant initial lab values.

**Table 2:** Significant initial lab values μL = microliters, g/dL = grams per deciliter, mmol/L = millimols per liter, mg/dL = milligrams per deciliter

Measurement	Value	Reference range
White blood cell count	5,500/μL	4,000-11,200/μL
Platelet count	268,000/uL	150,000-450,000/μL
Hemoglobin	13.2 g/dL	13.8-17.2 g/dL
Prothrombin time	13.1 seconds	11.0-15.0 seconds
Activated partial thromboplastin time	24.8 seconds	22.0-38.0 seconds
Sodium	133 mmol/L	136-145 mmol/L
High-density lipoprotein cholesterol	30 mg/dL	>40 mg/dL
Low-density lipoprotein cholesterol	111 mg/dL	<100 mg/dL
Triglycerides	217 mg/dL	14-199 mg/dL
Blood glucose	169 mg/dL	80-120 mg/dL
Lactate	1.5 mmol/L	<2.2 mmol/L

Hemoglobin dropped from 13 gm/dL to 10 gm/dL on day two but returned to baseline on day three. The lipid profile and blood glucose were noted to be poor as he missed a dose of insulin and he was not taking cholesterol-lowering medications. Other lab values not mentioned in Table 2, including electrolytes, renal function, liver function, and cardiac biomarkers, were all within normal limits.

CT studies, including abdominal, were carried out immediately. CT without contrast was complicated by the patient's movement and was labeled nondiagnostic, but sufficient to rule out hemorrhage. CTP similarly found no penumbra or core infarct to suggest ischemic stroke but was also labeled nondiagnostic due to artifact. CTA did not reveal any arterial occlusion or stenosis, which further supported that there was no acute infarction. Abdominal and pelvic CT with contrast did not reveal any acute findings.

**Figure 2:**
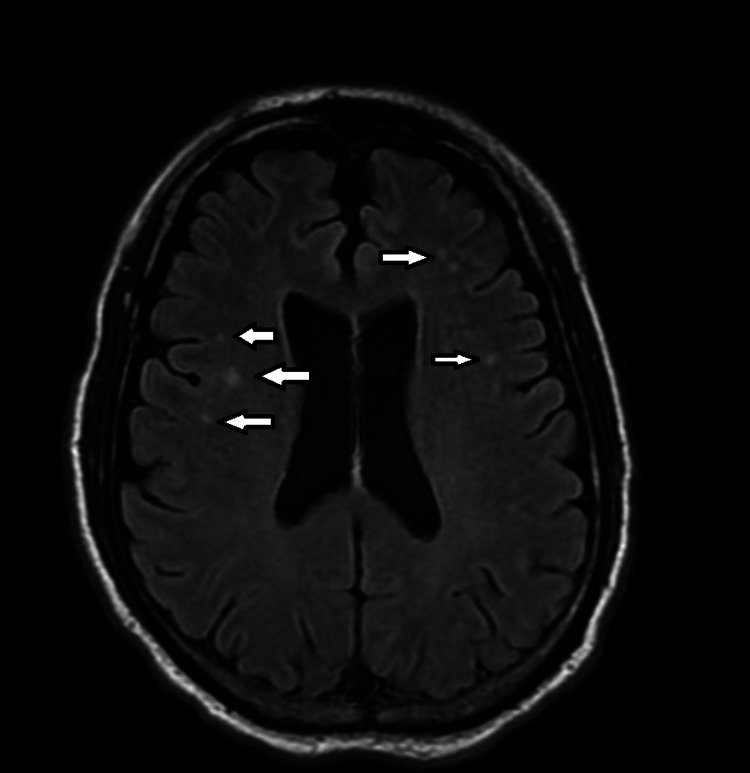
T2 FLAIR MRI without contrast showing punctate hyperintensities (white arrows) distributed in the deep cortical regions, 2024. This distribution is more consistent with changes seen in migraine than age-related changes, which are most often periventricular.

Hours later, T2 FLAIR MRI without contrast revealed several punctate hyperintensities at the gray-white junction bilaterally (see Figure 2). The radiologist's interpretation said this could represent age-related changes or are sequela of migraine headaches. As mentioned previously, these white matter changes appear in the deep cortex and not the periventricular regions, which is more consistent with migraine than age-related changes. By this time, TIA was considered unlikely as hours had passed. A digital rectal exam (DRE) was negative for gross blood. Unfortunately, the sample for occult blood testing was lost. Repeat testing was not ordered because the patient's hemoglobin stabilized after day two, serial blood counts were normal, and there was no melena or hematemesis observed in the hospital. The infectious disease workup was entirely negative.

On day 3, there was a small concern for leukemic infiltration of the CNS, but an MRI with gadolinium showed no abnormal enhancements. Lumbar puncture was also planned but deferred due to the negative MRI and continued clinical improvement. An EEG was deemed abnormal due to intermittent underlying bifrontal delta wave slowing but showed no epileptiform patterns. This finding was not conclusive and did not support or contradict epilepsy.

Therapeutic intervention and outcome

After the initial CT studies, a tissue plasminogen activator was held due to concern for gastrointestinal (GI) bleeding. By this time, the ischemic stroke had been essentially ruled out; otherwise, fibrinolysis or thrombectomy may have been considered more strongly. Fentanyl and ondansetron were given at this time for abdominal pain and nausea. Pantoprazole was given for prophylaxis against GI bleeding, atorvastatin due to stroke risk, levetiracetam due to possible seizure, and intravenous normal saline for maintenance.

Several hours after the previous interventions, and a complete history and physical, HM versus TP was the primary differential. He was started on ketorolac, diphenhydramine, and metoclopramide. It should be noted ketorolac was deemed appropriate only after concern for active GI bleed had been weakened by lack of acute findings on abdominal CT, nonbloody emesis during the examination, and negative DRE. Triptans and ergotamines were avoided in this patient due to the vascular nature of HM, in their absence this combination was chosen. His at-home insulin regimen was resumed. Dasatinib was held until day three as there was a possibility this was the source of the GI bleeding. Ultimately, it appeared that PUD was the source of bleeding as pantoprazole immediately controlled it. This would align with his prior episodes of hematemesis and melena that were due to PUD, years before he began taking dasatinib.

The patient was discharged on hospital day six on metoprolol for migraine prophylaxis, insulin, dasatinib, and levetiracetam at a low dose of 500 mg twice daily. This was a carefully made decision due to his history of provoked seizures (DKA, leukocytosis) and the current episode of a possibly unprovoked seizure, if one does not accept that pain and GI bleeding can be a trigger. The patient agreed the benefit of this well-tolerated drug outweighed the risks while outpatient neurology investigated his risk of future seizures.

A follow-up call confirmed he regained complete function and followed outpatient neurology as a migraine patient. They prescribed rimegepant for migraine abortion. Laboratory tests for HM, hyperviscosity, and hypercoagulability were ordered but notcompletede at the time of this writing.

## Discussion

Through critical reasoning, a patient with stroke-like symptoms who was previously defined as a TIA patient due to CML or TP patient due to provoked seizures was more reasonably characterized and treated as a sporadic HM patient. The first differential to be eliminated in this case was stroke as imaging results were negative, followed by TIA as symptoms persisted. TP was thought less likely than HM through a combination of imaging, clinical reasoning, and medical history. Even though TP could not be entirely ruled out, HM had more evidence in its favor as discussed below.

CVA is inconsistent in a patient presenting with flaccid tone, areflexia, and negative Babinski's sign whose symptoms fluctuated and lasted five days without any imaging findings or residual deficits [1,3]. Regardless, the patient's comorbidities place him at high risk for CVA and it was an important consideration [8]. T2DM, HTN, and HLD are all modifiable risk factors for stroke according to the American Heart Association [8]. Although CML itself is not a traditional risk factor [8], hypercoagulability [9] or paraproteinemia due to CML [10] can increase the risk for CVA. Further workup was ordered outpatient to investigate these states. Tyrosine kinase inhibitors like dasatinib have been associated with arterial ischemic events, but this drug in particular is more strongly associated with bleeding caused by platelet dysfunction [11]. The leukostasis argument for TIA given in July 2020 (see Figure 1) is weakened by normal white blood cell count and well-controlled CML during this presentation. For the current presentation, TIA is especially inconsistent given the time period of five days [4]. Trivial ischemia is also enough to trigger a migraine [7], and the investigators in 2020 noted that leukostasis causing CNS deficits in his case was irregular because he had leukocytosis but was not in a blast crisis. As noted previously, they had found the same deep cortical white matter hyperintensities in 2020 as seen in 2024, which may mean that this case was not a TIA but an HM [7]. Classic clinical evidence also points against an ischemic etiology, the pattern and nature of the FNDs presented are not related to any well-known vascular domain syndromes [3].

TP, as presumed in May 2020 (see Figure 1), is a possible explanation in a patient whose symptoms lasted five days after a witnessed seizure [4,5]. However, several findings make this unlikely. For one, the increased signal in the ictal area on MRI or other transient imaging findings is expected following a seizure [1,12]. Increased blood lactate levels are also often increased following a seizure, but this patient's lactate levels were normal [5,12]. The abnormal EEG findings of delta slowing are not conclusive for epilepsy, and this finding cannot differentiate between migrainous origin, TIA, and epilepsy in patients with FNDs [13]. It is also possible that if this patient had a seizure, this triggered a post-ictal migraine [5].

It is uncertain whether or not this patient has epilepsy; each possible seizure episode has been provoked and none were characterized in detail. The first episode was provoked in the context of DKA, the second in the context of leukocytosis and tumor lysis, and the third with bleeding from a peptic ulcer causing severe pain. This patient has never had a truly unprovoked seizure, which is necessary to diagnose epilepsy [12]. The misdiagnosis of status epilepticus, a prolonged and sometimes medically unresponsive chain of seizures, in cases of prolonged psychogenic non-epileptic seizures (PNES) is not uncommon [14]. Recall that the first seizure episode during DKA was sustained, repeated, and required multiple doses of lorazepam to control. The second possible episode was only characterized as unresponsiveness followed by weakness and was more likely attributed to a leukostatic picture. There is no explanation as to why this may have been a seizure. The third episode described a picture that may have been convulsive syncope in a patient with volume loss from diarrhea [15] but also may have been the patient's first unprovoked seizure if one considers the trigger of pain and GI bleeding insufficient. It was ultimately accepted that these episodes should be considered seizures because of the possible harm if untreated, but the diagnosis of epilepsy is difficult and prone to error [12,14,15]. This patient is definitely prone to provoked seizures and possibly suffers from epilepsy [12].

Sporadic HM is a reasonable explanation for the patient's presentation. The patient meets the criteria for the diagnosis of HM given in the International Headache Classification Disorders III (ICHD-III) edition, provided in Table 3 [16]. The case is sporadic given no family history [16].

**Table 3:** Diagnostic criteria for hemiplegic migraine according to the International Headache Classification Disorders III (ICHD-III) Reference: [16]

Diagnostic criteria for 1.2.3: Hemiplegic migraine	Patient
A. Attacks fulfilling criteria for 1.2 Migraine with aura and criterion B below	A. Patient meets both criteria only through historical extrapolation
Diagnostic criteria for 1.2: Migraine with aura.	
1. At least two attacks fulfilling criteria 2 and 3	1. Three attacks total
2. One or more of the following fully reversible aura symptoms:	2. The patient had the following aura symptoms:
Visual	Visual loss in all three episodes
Sensory	Sensory loss in 2024
Speech and/or language	No speech or language deficits
Motor	Motor weakness in all three episodes
Brainstem	Dysarthria in the 2024
Retinal	Scotoma in all three episodes
3. At least three of the following six characteristics:	3. The patient had the following characteristics:
At least one aura symptom spreads gradually over ≥5 minutes	Unknown spreading time
Two or more aura symptoms occur in succession	Symptoms occurred in succession, beginning with motor weakness and followed by sensory loss, scotoma, and headache in July 2024. Unknown progression in other cases.
Each individual aura symptom lasts 5-60 minutes	Symptoms lasted much longer in May 2020 and July 2024, unknown duration in August 2020
At least one aura symptom is unilateral	Symptoms were right-sided in all three cases
At least one aura symptom is positive	Scotoma of blue dots is a positive symptom in all three cases
The aura is accompanied, or followed within 60 minutes, by headache.	Symptoms were accompanied by headaches in all three incidents
4. Not better accounted for by another ICHD-3 diagnosis	4. The patient's presentation is not better accounted for by another ICHD-3 diagnosis
B. Aura consisting of both of the following	B. Aura consisted of both of the following in all three cases:
C. Fully reversible motor weakness	C. Fully reversible motor weakness
D. Fully reversible visual, sensory, and/or speech/language symptoms	D. Fully reversible visual and/or sensory symptoms

One must consider at least one of the prior two episodes of hemiparesis in fact having been an HM, and this is only possible if one has faith in the patient's recollection of the events as hospital records were sparse. Two other findings directly support this diagnosis. The migraine cocktail was effective in symptomatic relief, and MRI found changes in the deep white matter tracts that could represent age-related changes or sequela of migraine headaches [1,7]. Because the lesions were found in the patient's deep cortical tissue and not elsewhere, it was thought these were more likely related to migraine than to aging [7]. Ultimately the case meets the diagnostic criteria for HM, but the unique feature is the unexpected fluctuation in severity.

Migrainous aura has a complex pathophysiology relating to neuroinflammation, hyperexcitability, and ischemia. Ominously, MA is associated with a twofold increased relative risk for stroke [7]. The phenomenon of cortical spreading depression (CSD) has become largely accepted as the cause of aura; functional MRI (fMRI) studies have shown the pattern of CSD propagation to correspond with the movement of scintillating scotoma through the visual fields [7,17]. It is marked by cortical neuronal and glial depolarization propagating cephalad from the occipital cortex, accompanied by hypoperfusion lasting hours [17] with up to 12-17% reduction in cerebral blood flow during aura [18]. Other cortical origins have been observed as well, and it seems the area of CSD can explain even motor weakness [18]. This repeated ischemia may explain the increased burden of white matter lesions seen in migraineurs [7] and has even been proposed as the culprit for their increased stroke risk [18]. Several procoagulants, such as fibrinogen and high-sensitivity C-reactive protein, are increased in migraineurs as well, and there is a direct relationship between levels of these biomarkers and aura frequency [7,18]. Inflammatory biomarkers warrant a discussion of their own. Other than stroke, HM specifically has been associated with epilepsy based on the coexistence of these conditions in cases with shared ion channel mutations [19]. In knock-in mice with a certain calcium channel mutation (S218L, *CACNA1A*), it has been observed the threshold for CSD is especially low; these subjects are susceptible to repeated CSD in response to a single stimulus and often have coexisting HM and seizures [19]. Patients with this mutation also have especially prolonged CSD and severe aura symptoms [19]. Repeated CSD has been proposed as the mechanism of lowered seizure thresholds in patients with this mutation [19] and this may explain why our patient has had multiple provoked seizures in the past.

There is no specific literature to support this, but repeated CSD may also explain why symptoms fluctuated in this patient. Two major points must be accepted: aura and headache are two connected, but separate mechanisms [6,7,18], and non-steroidal anti-inflammatory drugs (NSAIDs) like ketorolac can prevent and abort migraine but have no effect in treating aura [6,18]. It can be theorized that the migraine abortive cocktail ended the headache and parietal CSD subsided, which may explain the patient's rapid motor and sensory improvement in 24 hours. It is proposed that CSD persisted in the occipital lobe, explaining the persistence of blurry vision and scotoma. The persistent CSD may have triggered spreading depolarization in the parietal lobe once more, but since the patient was already loaded with ketorolac, there was no headache. In the absence of literature investigating fluctuations of neurological symptoms in patients with HM, this is a possible explanation.

## Conclusions

The purpose of this case is to display an irregular sporadic HM patient, and raise curiosity of the relationship between FNDs in migraines and the mechanism of aura: CSD. With the mechanisms of aura described and the FNDs seen in this case, questions are raised about how exactly CSD produces FNDs. Furthermore, how is it that these symptoms fluctuate? Although further discussion is beyond the scope of this case report, this relationship does merit further research. Such studies may observe patients during an episode of HM and compare the cortical areas of CSD to physical FNDs using fMRI. Special attention should be paid if fluctuations in aura intensity can be invoked by aborting the migraine with NSAIDs or other medications. Ultimately, this may pave the way to finding a medication that can target aura in patients with especially debilitating symptoms as in HM.
